# Evaluation of Penalized and Nonpenalized Methods for Disease Prediction with Large-Scale Genetic Data

**DOI:** 10.1155/2015/605891

**Published:** 2015-08-04

**Authors:** Sungho Won, Hosik Choi, Suyeon Park, Juyoung Lee, Changyi Park, Sunghoon Kwon

**Affiliations:** ^1^Department of Public Health Science, Seoul National University, Seoul, Republic of Korea; ^2^Department of Applied Information Statistics, Kyonggi University, Suwon, Republic of Korea; ^3^Department of Biostatistics, Soonchunhyang University, College of Medicine, Seoul, Republic of Korea; ^4^Center for Genome Science, National Institute of Health, Osong Health Technology Administration Complex, Chungcheongbuk-do, Seoul, Republic of Korea; ^5^Department of Statistics, University of Seoul, Seoul, Republic of Korea; ^6^Department of Applied Statistics, Konkuk University, Seoul, Republic of Korea

## Abstract

Owing to recent improvement of genotyping technology, large-scale genetic data can be utilized to identify disease susceptibility loci and this successful finding has substantially improved our understanding of complex diseases. However, in spite of these successes, most of the genetic effects for many complex diseases were found to be very small, which have been a big hurdle to build disease prediction model. Recently, many statistical methods based on penalized regressions have been proposed to tackle the so-called “large P and small N” problem. Penalized regressions including least absolute selection and shrinkage operator (LASSO) and ridge regression limit the space of parameters, and this constraint enables the estimation of effects for very large number of SNPs. Various extensions have been suggested, and, in this report, we compare their accuracy by applying them to several complex diseases. Our results show that penalized regressions are usually robust and provide better accuracy than the existing methods for at least diseases under consideration.

## 1. Introduction

Accurate disease prediction is a central goal of clinical genetics, and much effort has been made to utilize the large-scale genetic data for a disease prediction model for complex disease. However, except for the fully penetrant Mendelian disorders, effect sizes of most disease susceptibility loci identified by genome-wide association studies (GWAS) are usually modest [1] and the presence of much larger number of genetic variants than the sample size (or so-called “large P and small N” problem) makes the construction of a disease risk prediction model intractable. For instance, the variation of predicted risk scores for each individual is proportionally related to the number of causal variants, and the accuracy of the predicted disease status decreases with the increase of the number of causal variants when the relative proportion of variance explained by causal variants is fixed [2, 3]. Also large P and small N problem prevents the estimation of the joint effect of all markers and thus prediction model building has been based only on marginal effects of variants [4–6]. Recently, various nonpenalized and penalized statistical methods have been suggested to tackle these issues. However, a comprehensive evaluation of existing methods has not been conducted yet.

The statistical methods for the disease risk prediction at the early stage were based on gene scores [4–6]. Causal variants often have additive effects on phenotypes, and a simple linear (logistic) regression can be adopted to estimate marginal effects of each variant under the assumption that there is no gene × gene and gene × environment interactions. Then, the coded genotypes of large-scale genetic data are multiplied with their marginal effect estimates and their sum for each individual can be incorporated to build the disease risk prediction model. Multiple studies showed that gene score-based approach is practically useful for building disease risk prediction [7]. This approach is computationally very efficient, and further extensions based on best linear unbiased predictor (BLUP) have been proposed in the literature [8–10]. However, for instance, if joint effects between multiple variants are substantial or there is large linkage disequilibrium between variants, the estimated gene score can be biased and the predicted disease risk becomes less accurate.

As an alternative to gene score-based approach, one may consider statistical learning methods in disease risk prediction. Statistical learning algorithms have been successful over the past decades in various learning tasks including text categorization, fraud detection, character and image recognition, natural language processing, and marketing. Disease risk prediction can be naturally posed as a classification problem, and support vector machines (SVMs) [11] and ensemble algorithms, in particular, random forests proposed by Breiman [12], have been often shown to yield more accurate predictions than other classification algorithms [13]. In particular, SVMs have been an important tool in classification because of their accuracy and flexibility in modeling different types of data. However, these methods have some drawbacks in disease risk prediction. Effects of variants on phenotypes in the prediction models from ensemble algorithms are difficult to interpret and SVMs do not provide class conditional probabilities [14]. Therefore, in this report, we focus on the penalized methods in logistic regression.

Recently, various penalized methods have been proposed to resolve the large P and small N problem. Examples include convex penalizations such as ridge [15–17] and LASSO [18] and nonconvex penalizations such as the smoothly clipped absolute deviation (SCAD) [19] and bridge [20]. In general, penalized methods have often provided more accurate predictions and easier interpretations than nonpenalized methods, especially when the number of samples is smaller than the number of variables. Some penalized methods automatically select relevant variables by setting the estimated coefficients of irrelevant variables as exactly zero. Also penalized methods enhance the accuracy of predictions by shrinking the coefficients of nonzero elements with data-adaptive tuning parameters.

In this report, we compare the performances of various nonpenalized and penalized methods in the prediction of diseases on data from Korea Association Resource (KARE) project that is a part of Korea Genome Epidemiology Study. We select individuals with extreme phenotypes among the participants in KARE project and consider the type 2 diabetes, obesity, hypertension, and three smoking-related phenotypes. The predictive performances of those nonpenalized and penalized methods are compared by area under the curve (AUC). Our results indicate that penalized methods tend to yield more accurate predictions than nonpenalized methods although their relative performances depend on particular diseases.

## 2. Methods

### 2.1. KARE Cohort

The KARE project, with 10,038 participants living in Ansung (rural) and Ansan (urban), was initiated in 2007 for large-scale GWAS based on the Korean population. Among the participants, 10,004 individuals were genotyped for 500,568 SNPs with the Affymetrix Genome-Wide Human SNP array 5.0. We discarded SNPs with *p*-values for Hardy-Weinberg equilibrium (HWE) less than 10^−6^, with genotype call rates less than 95%, or minor allele frequencies (MAF) less than 0.01, and 352,228 SNPs were left for subsequent analysis. Individuals with low call rates (<95%, *n* = 401), high heterozygosity (>30%, *n* = 11), gender inconsistencies (*n* = 41), or serious concomitant illness (*n* = 101) were excluded from analysis. We considered independent samples and excluded related or identical individuals whose computed average pairwise identical in state value was higher than that estimated from first-degree relatives of Korean sib-pair samples (>0.8, *n* = 608). In total, 8,842 individuals were analyzed. From randomly selected 20 duplicate samples, we found that genotype concordance rates exceeded 99.7%, with no single SNP excessively discordant. The population substructure was handled with EIGENSTRAT approach [21] and we chose 10 principal component scores. Missing genotypes were imputed with Beagle [22].

#### 2.1.1. Type 2 Diabetes (T2D)

T2D mainly occurs in people aged over 40, and it is diagnosed with level of glucose and hemoglobin a1c (hba1c) in blood. In our studies, individuals were selected as being affected with type 2 diabetes if their hba1c are larger than 6.5, fasting plasma glucoses are larger than or equal to 126, or 2-hour postprandial blood glucoses are larger than or equal to 200. In total, there were 1182 affected individuals, and 2364 individuals not satisfying the condition for type-2 diabetes and older than the other unaffected individuals were considered as controls. As environmental variables, we considered area (Ansan/Ansung), sex, age, body mass index (BMI), systolic blood pressure (SBP), diastolic blood pressure (DBP), triglyceride, and ten PC scores.

#### 2.1.2. Obesity

Obesity status was determined by BMI. Individuals were considered as cases if their BMIs are larger than 27, and there were 1022 affected individuals in KARE cohort. We also selected 2325 individuals with BMIs less than 27 and older than the other unaffected individuals as controls. We considered area, sex, age, height, waist-hip ratio, SBP, DBP, high density lipoprotein, triglyceride, and ten PC scores as environmental variables.

#### 2.1.3. Hypertension

Hypertension status was determined by SBP and DBP. 1035 individuals with SBPs and DBPs larger than 140 and 80, respectively, were considered as cases. 2290 individuals whose SBPs and DBPs were less than 120 and 80, respectively, were selected as controls. Environmental variables considered were area, sex, age, BMI, and ten PC scores.

#### 2.1.4. Cigarettes Smoked per Day (CPD)

For smoking-related phenotypes, we considered only male samples for predicting smoking behaviors because the number of female smokers was very small. CPD was defined to detect the nicotine dependence of each individual. Individuals whose number of cigarettes per day was larger than 20 were defined as being addicted to nicotine, and 333 individuals were selected as cases. Individuals were chosen as controls if the number of cigarettes per day was less than 10, and 375 males were chosen as controls. Environmental variables were area, age, BMI, waist-hip ratio, triglyceride, SBP, and ten PC scores.

#### 2.1.5. Smoking Initiation (SI)

Smoking status for each individual has four categories: never smoked, former smoker, occasional smoker, and habitual smoker. Males who never smoked were defined as controls, and males who occasionally or habitually smoke were defined as cases. There were 3357 cases and 807 controls, and the same clinical variables as in CPD were environmental variables.

#### 2.1.6. Smoking Cessation (SC)

SC was defined with smoking status as SI, but we used different categories for cases and controls. Males who never smoked were defined as controls, and males who occasionally or habitually smoke or smoked before were defined as cases. The numbers of cases and controls are 2064 and 1293, respectively, and environmental variables were the same clinical variables as in CPD.

### 2.2. Disease Risk Prediction Model Building

#### 2.2.1. Notations

Let *y*_*i*_ be a dichotomous phenotype for individual *i*, and affected and unaffected individuals are coded as 1 and 0, respectively. The sample size is denoted as *n* = *n*_*a*_ + *n*_*u*_, where *n*_*a*_ and *n*_*u*_ denote the numbers of cases and controls, respectively. We assume that there are *p*_1_ genetic variants and *p*_2_ environmental variants including an intercept. Therefore the total number of variables is *p* = *p*_1_ + *p*_2_. **x**_*i*_ denotes a vector with *p* covariates for individual *i*, and the coded genotypes of the *k*th variant and the *l*th environmental variable were denoted by *x*_1*ik*_ and *x*_2*il*_, respectively. The coefficient vector of *p* covariates is denoted by **β**.

#### 2.2.2. Cross Validation

To see the effect of sample size, we selected *n* individuals where *n*_*a*_ cases and *n*_*u*_ controls with extreme phenotypes were chosen, and the relative ratios of *n*_*a*_ to *n*_*u*_ are assumed to be equal to their ratios between all available cases and controls in KARE cohorts. We evaluated the accuracies of the disease risk prediction models for different choices of *n*. Accuracies of the disease prediction models were assessed via 10-fold cross validation, and AUCs were calculated with 10 replicates. All individuals were randomly divided into 10 different subgroups with the same number of cases and controls. Each subgroup was used as test set once across ten replicates and therefore there is no overlap between test set in different replicates.

#### 2.2.3. Feature Selection and Risk Prediction

Numbers of available genetic markers seem to be related to the prediction accuracy, and different numbers of genetic variants were selected to build the disease risk prediction model. We choose the top *p*_1_ genetic variants by the order of *F*-ratio from train set. If we let ${\overset{-}{x}}_{1.k}^{\left(l\right)}$ be the average expression level of the *k*th variant for individuals with phenotype *l* and denote the overall mean expression level of the *k*th variant by ${\overset{-}{x}}_{1.k}$, the *F*-ratio of the *k*th variant [23, 24] is defined as

(1) $$\begin{array}{c} F_{k}=\frac{\sum_{i=1}^{n}\sum_{l=0}^{1}I\left(y_{i}=l\right){\left({\overset{-}{x}}_{1.k}^{\left(l\right)}-{\overset{-}{x}}_{1.k}\right)}^{2}}{\sum_{i=1}^{n}\sum_{l=0}^{1}I\left(y_{i}=l\right){\left(x_{1ik}-{\overset{-}{x}}_{1.k}^{\left(l\right)}\right)}^{2}}. \end{array}$$

Then we build the disease risk prediction model with those selected top *p*_1_ genetic variants and *p*_2_ environmental variables on train set and apply the prediction model to test set.

### 2.3. Nonpenalized Methods

#### 2.3.1. Genetic Risk Scores (GRS)

The marginal effects of covariates are tested with *F*-ratio [23, 24]. Then, the coded genotypes of significant variants at *α* = 0.05 level are summed to calculate GRS, and GRS and environmental variables were incorporated into the logistic regressions as covariates to build the final disease risk model on train set. The disease risk scores are calculated for individuals on test set and its accuracy of disease risk prediction model is evaluated.

#### 2.3.2. MultiBLUP

Polygenic effects explained by available SNPs can be modeled by the linear mixed model whose variance covariance matrix is parameterized with the genetic relationship matrix [25–27], and BLUP can be used to predict the disease risk by genetic effects. However, those approaches assume that effects of all SNPs are homogeneous in spite of their heterogeneity. For instance, it has been shown that MAFs of SNPs may reveal some information about genetic architecture [28] and random effects need to be defined for SNPs with different spectra of MAFs separately. MultiBLUP [10] categorizes each SNP into different classes with distinct effect sizes or linkage disequilibrium block and applies a linear mixed model with multiple random effects to improve the accuracy of the prediction model [10].

### 2.4. Penalized Methods

Various penalized methods have been recently proposed, and we consider five penalized methods in our comparison: ridge [29], LASSO [30], elastic-net [31], SCAD [32], and truncated ridge (TR) [33–35]. The *p* dimensional coefficient vector **β** = (*β*_1_,…, *β*_*p*_)^*t*^ can be estimated by minimizing the penalized negative log-likelihood:

(2) $$\begin{array}{c} \frac{1}{n}\overset{n}{\underset{i=1}{\sum }}\left\{-y_{i}x_{i}^{t}\beta +\log\left(1+\exp\left(x_{i}^{t}\beta \right)\right)\right\}+\overset{p}{\underset{j=1}{\sum }}J_{\lambda }\left(\left|\beta_{j}\right|\right), \end{array}$$

where *J*_*λ*_ is a penalty function and *λ* is a vector of tuning parameter that can be determined by a search on an appropriate grid. Each penalized regression requires the estimation of *λ*, and 100 grid points of *λ* were considered from “glmnet” function in *R* for all the methods.

#### 2.4.1. Ridge

In linear regression, estimates from least square method are quite unstable under severe multicollinearity because of their large variances. Ridge penalty

(3) $$\begin{array}{c} J_{\lambda }\left(t\right)=\lambda t^{2} \end{array}$$

was originally developed to stabilize the sample performance of least square estimates by shrinking their absolute values toward zero [29]. Ridge penalty controls the amount of shrinkage effect by choosing the tuning parameter *λ*, and the resulting ridge estimates tend to have a smaller variance than least square estimates. In particular, ridge regression can be conducted even when *p* is much larger than *n*, where the least square method does not have a model identifiability. However, ridge estimates have a drawback in the interpretation of the final model because all the covariates are included in the final model regardless of the choice of *λ*. Hence, ridge regression must be conducted together with an extra selection process such as stepwise subset selection or truncation methods.

#### 2.4.2. LASSO

LASSO was proposed by Tibshirani [30] to achieve both shrinkage and covariate selection via the penalty

(4) $$\begin{array}{c} J_{\lambda }\left(t\right)=\lambda t. \end{array}$$

LASSO selects relevant covariates and estimates their coefficients simultaneously by controlling the tuning parameter *λ* [30]. LASSO often shows a quite stable performance, especially when the sample size is small [32, 36], and achieves higher prediction accuracy than other penalized methods. LASSO has been applied to various statistical models such as Gaussian graphical models [37] because there are fast and efficient algorithms that are easily implementable [37–40]. However, several defects of LASSO have been reported in the literature [36, 41–43]. For example, LASSO tends to overfit, that is, selecting more covariates than expected [39], and is known to have a confliction between correct selection and optimal prediction [38]. To remedy such defects, modified versions of LASSO [36] were proposed and extended to the large P and small N problem [44].

#### 2.4.3. Elastic-Net

Elastic-net penalty proposed by Zou and Hastie [31] is a convex combination of LASSO and ridge penalty is

(5) $$\begin{array}{c} J_{\lambda }\left(t\right)=\lambda \left(at+\left(1-a\right)t^{2}\right). \end{array}$$

Here we considered 20 equally spaced grid points from zero to one for *a*. Elastic-net has more desirable properties than LASSO and ridge. For instance, ridge tends to keep all the covariates in the final model and hence is undesirable when there are many noncausal variants. In contrast, LASSO cannot select larger number of covariates than the sample size and tends to select a single covariate among highly correlated covariates. However, by choosing appropriate *λ* and *a*, elastic-net enables us to have balanced estimates, producing a slightly more complex model than LASSO but far simpler model than ridge. Also it achieves a grouping effect [30] on highly correlated covariates. However, elastic-net shares the disadvantage of LASSO; that is, it often overfits, which can be resolved by applying a data adaptive weight vector [45].

#### 2.4.4. SCAD

The SCAD penalty introduced by Fan and Li [32] is

(6) $$\begin{array}{c} \frac{\partial J_{\lambda }\left(t\right)}{\partial t}=\min\left\{\lambda ,\frac{{\left(a\lambda -t\right)}_{+}}{a-1}\right\}\;\text{for}\;\text{some}\;a>2, \end{array}$$

and *a* = 50 is used for our own optimization algorithm. SCAD has several desirable properties over LASSO [32]. First, SCAD produces the same unbiased estimates as usual nonpenalized estimates of the covariates selected by SCAD. Hence SCAD can be considered as a stable version of best subset selection [46], achieving a unique benefit of the unbiased coefficient estimate [32]. Second, SCAD is known to have the oracle property [32]; that is, the set of selected covariates are asymptotically equal to the set of true causal variants. However, in spite of theoretical optimality of SCAD [47], its estimates can be poor unless the sample size is large and the effects of signal covariates are strong. In addition, similarity between numerically estimated values and theoretical ones cannot be measured because of the nonconvexity of SCAD penalty, and the computational cost for SCAD is often much more expensive than LASSO.

#### 2.4.5. TR

As we mentioned above, ridge cannot be directly used in identifying important covariates. However, TR [35] can produce sparse estimates and inherits the same shrinkage effect as ridge that results in high prediction accuracy in the presence of multicollinearity [48]. To obtain TR estimates, we first obtain usual ridge estimates with tuning parameter *λ* and then truncate them with truncating level *a*. Hence TR declares the ridge coefficients whose absolute values smaller than *a* as zero and keeps the other large coefficients intact. An appropriate choice of truncating level enables us to identify a correct model while the final estimates still keep the same shrinkage property as ridge [33–35], and 20 grid points equally spaced in logarithmic scale from 0.01 to 0.001 were considered for *a*.

## 3. Results

**Figure 1:**
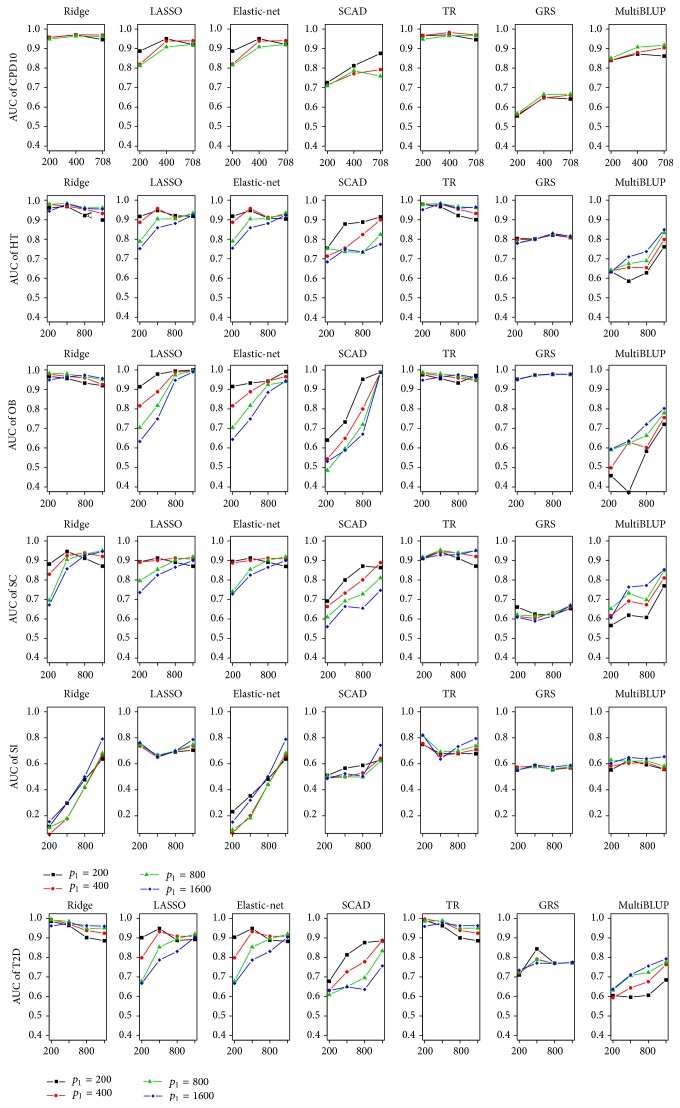
AUCs from test set. AUCs for T2D, obesity, hypertension, CPD10, SC, and SI from test set were calculated for different *n* and *p*_1_. TR indicates the truncated ridge.

To see the differences of penalized methods, we calculated AUCs of those methods on test set and the number of nonzero coefficients as a function of sample size.  Figure 1 shows that relative performance of each method substantially depends on phenotypes, and least AUCs are often observed for SI, followed by SC. Their least AUCs may be explained by the relative importance of genetic components for each phenotype. We calculated the relative proportion of variances, *h* ^2^, explained by genotyped variants with GCTA program [27, 49]. *h* ^2^ for binary traits was estimated with all available samples by using default options, and Table 1 shows estimates for *h* ^2^. In particular, the proportion between cases and controls for each phenotype is different from true prevalence, and the ascertainment bias often happens. However the performance of each method may be related to unadjusted estimates of *h* ^2^ and ascertainment bias was not taken into account. According to Table 1, the genotyped variants explain around 25% of phenotypic variances for hypertension and CPD. However the standard error of *h* ^2^ for CPD10 is large, and genetic components for all smoking-related phenotypes seem relatively less informative.

**Figure 2:**
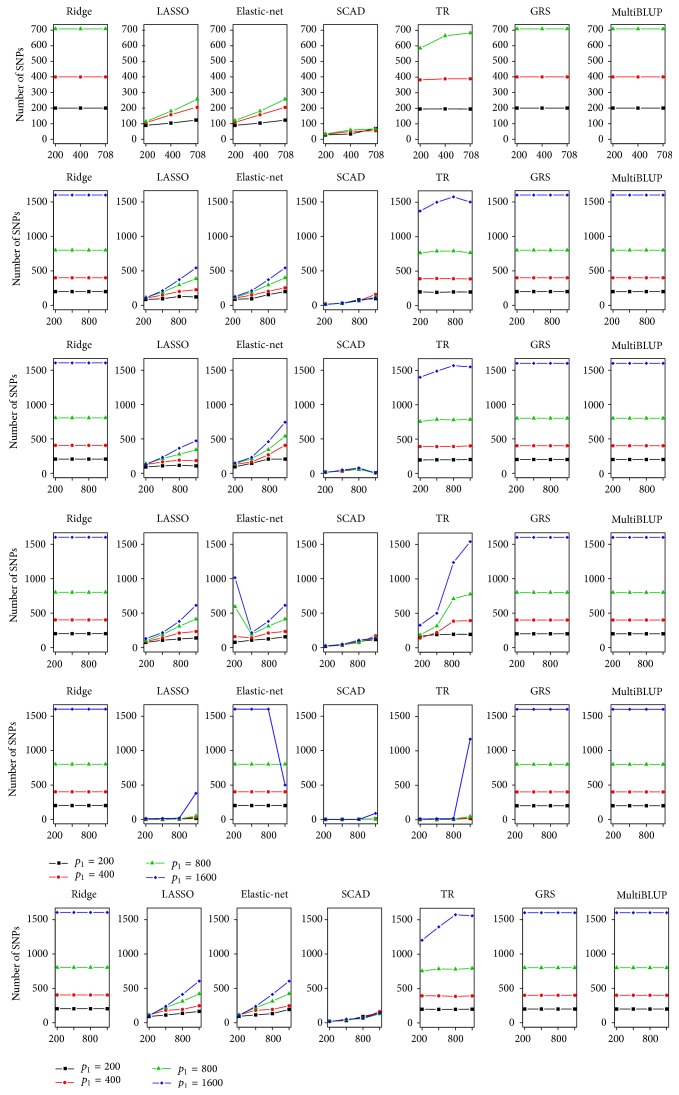
Number of nonzero *p*_1_ in the disease risk prediction model. Numbers of nonzero coefficients of SNPs in disease risk prediction model were provided for different *n* and *p*_1_. TR indicates the truncated ridge.

Figure 1 shows that AUCs of two nonpenalized methods, GRS and MultiBLUP, on test set were generally outperformed by the penalized methods across various levels of *n* and *p*_1_. Both approaches do not consider the joint effects among multiple causal SNPs. GRS assumes that effect sizes for causal SNPs are homogeneous and MultiBLUP assumes that sums of each SNP affect the normal distribution. However, penalized methods estimate individual effects of each SNP by shrinking each coefficient. This may explain the superiority of penalized methods over nonpenalized methods, but if those assumptions for nonpenalized methods are satisfied, they may perform better than penalized methods approaches. Interestingly MultiBLUP performs better than GRS except OB if *n* is larger, and AUC improvement of MultiBLUP for larger *n* is more substantial than GRS. Therefore, MultiBLUP seems to be more reasonable choice than GRS. Comparing overall performances of penalized methods, it can be seen that ridge and TR are the best, LASSO and elastic-net are the second, and SCAD is the last even though the performance of each method depends on specific diseases and the levels of *n* and *p*_1_. Regardless of *n* and *p*_1_, ridge was the best performer even for small *n* for all phenotypes except SI. For SI, it seems that the performance of ridge depends on *n* rather than *p*_1_. TR virtually has almost the same prediction accuracy, and Figure 2 shows that its model complexity is similar with ridge for CPD, obesity, hypertension, and T2D. This observation is also strengthened by the fact that the optimal value of truncation parameter, *a*, is around 0.001, and thus the effect of truncation parameter on model complexity is almost negligible for these data sets. However, Figure 1 shows that differences between ridge and TR are substantial for SC and SI. AUCs of TR depend on *p*_1_ and, in particular, are large even when *n* is small, which indicates that AUCs of TR depend less on *n* than ridge. Robustness of TR can be partially explained by smaller model complexity than ridge in Figure 2. For instance, TR usually selects quite small number of SNPs (at most 15.3 SNPs for *n* ≤ 800 and 46.6 SNPs for *p*_1_ ≤ 800) but achieves higher prediction accuracy than ridge when *n* is less than 800. However, when *p*_1_ = *n* = 1600, TR selects the same number of SNPs as ridge. Thus, we can conclude that the effect of truncation parameter diminishes for large *n*, which explains higher prediction accuracy when *n* is small.

LASSO and elastic-net show relatively large dependency on *n* and *p*_1_ in prediction accuracy and model complexity for whole phenotypes except SI, and their AUCs are proportionally related to *n* but inversely related to *p*_1_. Although their prediction accuracies are lower than those of ridge and TR for small *n*, they perform as well as ridge with small numbers of SNPs for large *n*. For instance, LASSO includes about 100 SNPs for *n* = 1600 and *p*_1_ = 200 and about 500 SNPs for *n* = 1600 and *p*_1_ = 1600, which indicates that we can construct prediction models without using the whole SNPs. Elastic-net tends to behave quite similarly as LASSO and it selects slightly larger number of SNPs for whole phenotypes except SI.

In terms of model complexity, there are substantial differences among penalized methods. Figure 2 show that SCAD selects the smallest number of covariates, while other methods such as LASSO and elastic-net usually include much more covariates. Ridge always includes all covariates, and model complexity for TR depends on data. However, even though SCAD generates the simplest model, SCAD is less preferable if it achieves the least performance among penalized regressions. For *n* = 1600, SCAD performs as well as other methods while still keeping small number of SNPs. For instance, for obesity, AUCs of SCAD are virtually the best and select extremely sparse models that have only 7.3 and 3.4 SNPs for *p*_1_ = 200 and *p*_1_ = 1600, respectively. Therefore, we can conclude that SCAD is appropriate as long as relatively large number of individuals is available.

## 4. Discussion

In this study we have considered five penalized and two nonpenalized statistical methods with six case-control datasets that are computationally feasible at the genome-wide scale. Each method was utilized to build the disease risk prediction model with different sample sizes and numbers of variants, and the accuracy of disease risk prediction models was evaluated with cross validation. Cross validation tends to overestimate the prediction accuracy, and results should be interpreted with care. A more reliable but time-consuming way is to compare the methods on random partitions of data. However cross validation does not have a strong preference towards a specific method and it may give us a rough idea on prediction accuracies of methods. According to our results, dense methods such as ridge and TR are usually more accurate than sparse methods such as LASSO and SCAD. For a large sample size, prediction accuracies from penalized methods are expected to be similar to that from ridge [23, 35, 50].

However, in spite of our comprehensive evaluations, various factors such as filtering conditions for SNPs or individuals, test statistic for prescreening, and ways of obtaining tuning parameters can affect the accuracy of the final risk prediction model, and depending on their choices, accuracy of disease risk prediction model can be substantially different. In this context, the 1-standard deviation rule [14] for tuning parameters was adopted to reduce overfitting problem. However, it did not provide any significant improvement in the results, which may indicate that there may be many causal genetic variants with small effects in the analyzed data sets. This consistently explains the reason why dense methods outperformed sparse methods such as LASSO and SCAD in our analysis. Moreover, while the results from SCAD were quite unstable for *a* = 10, the choice of *a* = 50 led to the better prediction accuracy. These findings suggest that most of SNPs have a small causal effect on diseases considered in this report. In this sense, sparse methods such as SCAD may not be preferred for infinitesimal model [51] unless the sample size is sufficiently large.

In this report we have compared various penalized regression methods. However, we have not considered more recent methods such as bootstrapping methods [33, 52, 53]. Most of them usually suffer from intensive computational burden induced by tuning extra parameters such as bootstrap size, and thus they are not computationally feasible at genome-wide scale. Alternatively, in the follow-up studies, we pursue the direction of refining the penalized methods considered in this report because there is still a significant room for improvement.

## Supplementary Material

Figure 1: AUCs from train set for T2D, obesity and hypertension AUCs for T2D, obesity and hypertension from train set were calculated for different *n* and *p*1 TR indicates the truncated ridge.
Figure 2: AUCs from train set for CPD, SC and SI. AUCs for CPD, SC and SI from train set were calculated for different *n* and *p*1. TR indicates the truncated ridge.

## Figures and Tables

**Table 1:** Relative proportion of variance explained by genotyped SNPs.

	T2D	Obesity	Hypertension	CPD	SI	SC
*h* ^2^	0.147276	0.14922	0.296246	0.243554	0.052088	1.00*E* − 06
*σ*(*h* ^2^)	0.097091	0.10029	0.100675	0.424123	0.080256	0.102595
