# Supplementary material for: Evaluation of Penalized and Nonpenalized Methods for Disease Prediction with Large-Scale Genetic Data
Source: Biomed Res Int. 2015 Aug 4;2015:605891. doi: 10.1155/2015/605891 (PMC4539442; doi:10.1155/2015/605891)

Web-based Supplementary Materials for "Evaluation of penalized and non-penalized methods for disease prediction with large-scale genetic data" by

Sungho Won[1‡], Hosik Choi[2‡], Sooyeon Park[3], Juyoung Lee[3],

Changyi Park[4§], Sunghoon Kwon[5§]

[1]Dept of Public Health Science, Seoul National University, Seoul, South Korea

[2]Dept of Applied Information Statistics, Kyonggi University, Suwon, South Korea

[3]Center for Genome Science, National Institute of Health, Osong Health Technology Administration complex, Chungcheongbuk-do, Seoul, Korea

[4]Dept of Statistics, University of Seoul, Seoul, South Korea

[5]Dept of Applied Statistics, Konkuk University, Seoul, South Korea

[‡]These authors contributed equally to this work

[§]Corresponding to

Changyi Park, Dept. of Statistics, University of Seoul, South Korea

(Email) park463@uos.ac.kr, (Tel) +82-2-6490-2634

Sunghoon Kwon, Dept of Applied Statistics, Konkuk University, Seoul, South Korea

(Email) shkwon0522@konkuk.ac.kr, (Tel) +82-2-450-0532

Figure 1. AUCs from train set for T2D, obesity and hypertension AUCs for T2D, obesity and hypertension from train set were calculated for different n and p_1. TR indicates the truncated ridge.

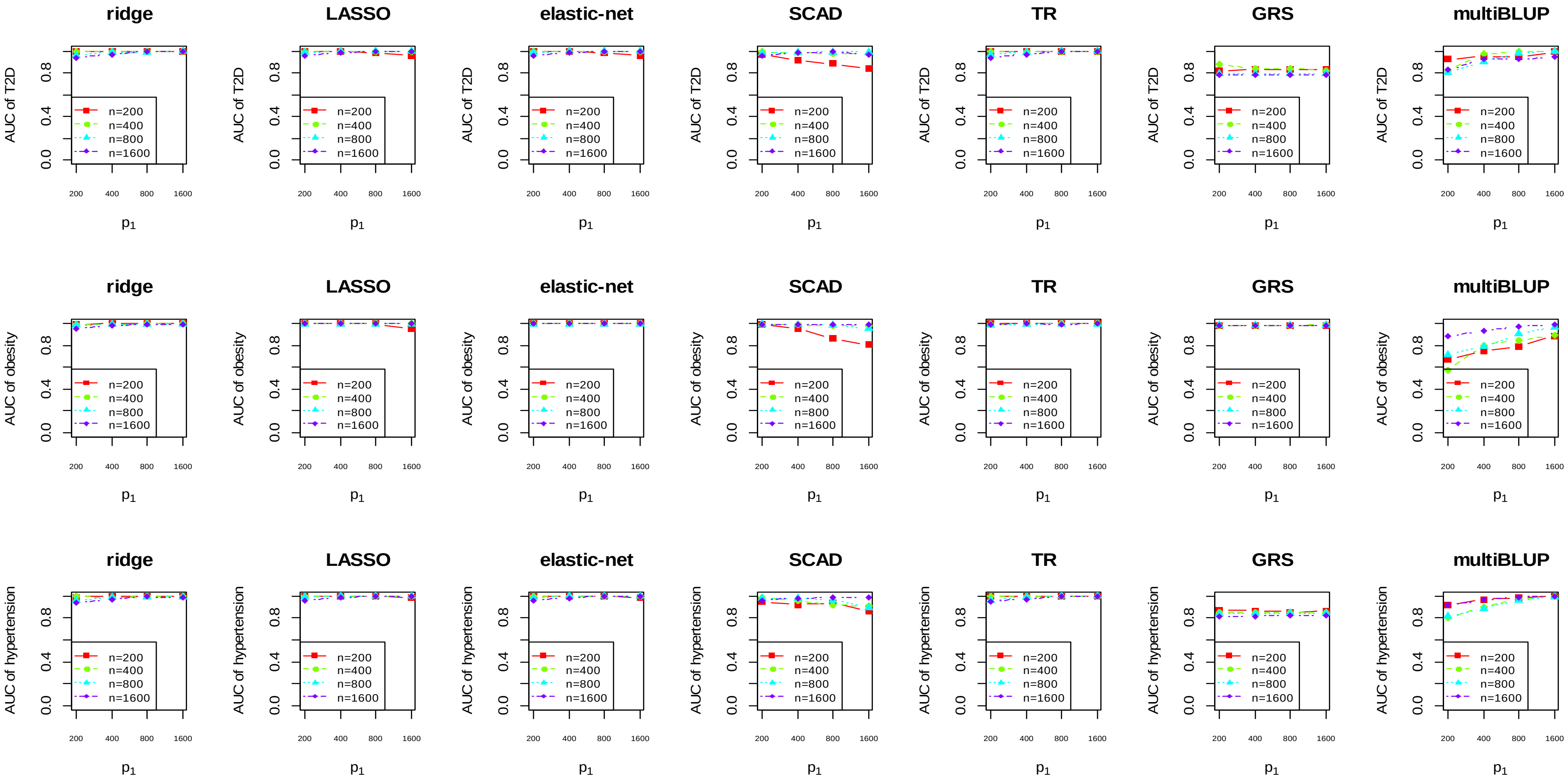

Figure 2. AUCs from train set for CPD, SC and SI. AUCs for CPD, SC and SI from train set were calculated for different *n* and p_1. TR indicates the truncated ridge.

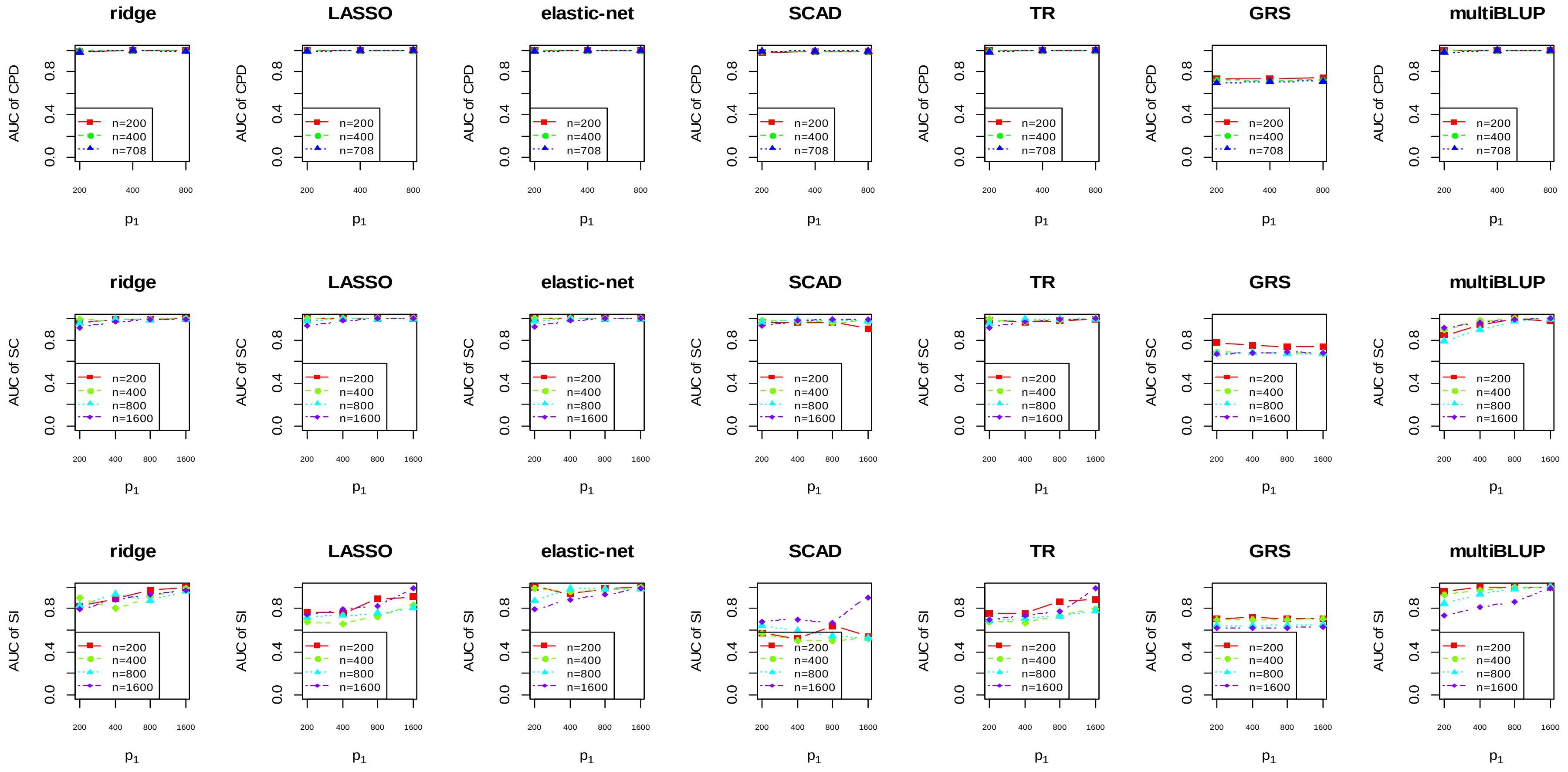

Supplement: Supplementary file 1 — Figure 1: AUCs from train set for T2D, obesity and hypertension AUCs for T2D, obesity and hypertension from train set were calculated for different n and p1 TR indicates the truncated ridge. Figure 2: AUCs from train set for CPD, SC and SI. AUCs for CPD, SC and SI from train set were calculated for different n and p1. TR indicates the truncated ridge. [file 605891.f1.pdf]
